# Ischemic Neuroprotection by Insulin with Down-Regulation of Divalent Metal Transporter 1 (DMT1) Expression and Ferrous Iron-Dependent Cell Death

**DOI:** 10.3390/biom14070856

**Published:** 2024-07-15

**Authors:** Francesca Fenaroli, Alessandra Valerio, Rosaria Ingrassia

**Affiliations:** 1 Section of Pharmacology, Department of Molecular and Translational Medicine, University of Brescia, 25123 Brescia, Italyalessandra.valerio@unibs.it (A.V.); 2 Section of Biotechnologies, Department of Molecular and Translational Medicine, University of Brescia, 25123 Brescia, Italy

**Keywords:** in vitro ischemia, insulin neuroprotection, oxygen glucose deprivation (OGD), divalent metal transporter 1 (DMT1), ferroptosis, neurodegeneration

## Abstract

**Background:** The regulation of divalent metal transporter-1 (DMT1) by insulin has been previously described in Langerhans cells and significant neuroprotection was found by insulin and insulin-like growth factor 1 treatment during experimental cerebral ischemia in acute ischemic stroke patients and in a rat 6-OHDA model of Parkinson’s disease, where DMT1 involvement is described. According to the regulation of DMT1, previously described as a target gene of NF-kB in the early phase of post-ischemic neurodegeneration, both in vitro and in vivo, and because insulin controls the NFkB signaling with protection from ischemic cell death in rat cardiomyocytes, we evaluated the role of insulin in relation to DMT1 expression and function during ischemic neurodegeneration. **Methods:** Insulin neuroprotection is evaluated in differentiated human neuroblastoma cells, SK-N-SH, and in primary mouse cortical neurons exposed to oxygen glucose deprivation (OGD) for 8 h or 3 h, respectively, with or without 300 nM insulin. The insulin neuroprotection during OGD was evaluated in both cellular models in terms of cell death, and in SK-N-SH for DMT1 protein expression and acute ferrous iron treatment, performed in acidic conditions, known to promote the maximum DMT1 uptake as a proton co-transporter; and the transactivation of 1B/DMT1 mouse promoter, already known to be responsive to NF-kB, was analyzed in primary mouse cortical neurons. **Results:** Insulin neuroprotection during OGD was concomitant to the down-regulation of both DMT1 protein expression and 1B/DMT1 mouse promoter transactivation. We also showed the insulin-dependent protection from cell death after acute ferrous iron treatment. In conclusion, although preliminary, this evaluation highlights the peculiar role of DMT1 as a possible pharmacological target, involved in neuroprotection by insulin during in vitro neuronal ischemia and acute ferrous iron uptake.

## 1. Introduction

Divalent metal transporter 1 (DMT1) (Nramp2/SLC11A2, solute carrier family 11 member 2) [1,2], the major importer of ferrous iron, is responsible for the uptake of non-transferrin-bound iron transport (NTBI) and divalent metals, in equilibrium with the receptor-mediated endocytosis of transferrin-bound iron (TBI) and dietary iron transported to peripheral tissues, after uptake through the apical membrane of the enterocytes in the acidic duodenal milieu [3]. The duodenal NTBI absorption of iron and heavy metals is influenced by systemic iron homeostasis, also regulated by inflammation [4], that may lead to iron dyshomeostasis, accumulation in peripheral tissues, and the possible development of neurodegenerative diseases. DMT1 is widely expressed throughout the rat brain, particularly in the olfactory bulb, hippocampus, cerebellum, and ventral area [1], and is up-regulated in several neurodegenerative diseases with the alteration in the intracellular transport of heavy metals [5]. DMT1 is finely regulated at the transcriptional level by the 5′ and 3′ alternative splicing. The 5′ prime splicing drives the expression of the alternative promoters 1A and 1B, the latter responsive to NF-kB [6], and the 3′ alternative splicing leads to the expression of alternative transcripts, with or without an iron-responsive element (IRE), inducing the post-transcriptional regulation secondary to intracellular iron levels [7]. The four alternatively spliced products of DMT1 mRNA isoforms are thus 1A(+)IRE, 1A(−)IRE, 1B(+)IRE, and 1B(−)IRE according to the 5′ and 3′ splicing.

It is noteworthy that neuroinflammation represents the prodromal event for several neurodegenerative diseases, like Parkinson’s and ischemia, with the involvement of the NFkB signaling and the NFkB-dependent activation of DMT1 [8,9,10,11]. Interestingly, DMT1 regulation by insulin was described in Langerhans cells [12], and, in insulin-producing cells, the NF-kB/p65 DNA binding was reduced by small-molecule inhibitors of HDAC lysine deacetylase, leading to the abrogation of the inflammation-derived NF-kB transcriptional activation, with the protection of β-cells from inflammation-induced apoptosis [13]. Again, in the mouse pancreatic beta cell line MIN6, the over-expression of the fat mass and obesity-associated (FTO) gene, highly expressed not only in the pancreas but also in the CNS, inhibits insulin secretion with the activation of NF-kB, ROS production, and pancreatic β-cell dysfunction, thus sustaining the increased risk of obesity and the development of type 2 diabetes [14]. In this regard, further evidence showed the activation of the NFkB-inducing kinase (NIK) in islet β cells with their consequent failure, as established in obesity, associated with diabetes, also induced in a mice model at a young age [15].

Importantly, both insulin mRNA and insulin were detected in the CNS, in several regions, including the hypothalamus, hippocampus, cerebral and cerebellar cortex, and olfactory bulb with specific roles of neuroprotection, the control of growth hormone secretion, and the regulation of appetite and neuronal glucose uptake; and the impairment of insulin signaling has been associated with memory decline in humans [16,17]. Acute exposure to insulin was found to influence dopamine-related transmission in basal ganglia with the consequent activation of striatal insulin receptors that amplifies axonal dopamine release in rodent brain slices, although mediated by the indirect transmission with increased striatal cholinergic interneuron excitability and enhanced dopamine release via nACh receptors on dopaminergic axons that express InsRs [18].

It is noteworthy that, in this relationship, significant neuroprotection was found by insulin and insulin-like growth factor 1 during neurodegeneration in models of experimental cerebral ischemia by intranasal treatment, as a safe and effective route of administration, bypassing the blood–brain barrier and maximizing the distribution to the central nervous system (CNS) without systemic side effects and metabolic influence [19]. Indeed, insulin and insulin-like growth factor 1 (IGF-1) have been shown to play a key role in the CNS by regulating neuronal growth and plasticity with multiple effects in the brain, including neuroprotection in cerebral ischemia and stroke patients [20]. Moreover, the intranasal treatment with a low dose of insulin in a rat model of Parkinson’s disease, after a 6-OHDA-induced lesion, lasting for 14 days determined a significant decrease in the 6-OHDA-induced motor dysfunction and dopaminergic cell death [21]. More interestingly, the direct relationship between insulin and NFkB signaling during ischemia was found in rat cardiomyocytes, where insulin was shown to target the NFkB signaling with protection from ischemic cell death [22]. It is noteworthy that the convergence of the insulin and leptin signaling pathways on the IRS/PI3K/Akt axis was previously described in midbrain dopaminergic neurons [23]. In this regard, leptin neuroprotection after an ischemic neuronal injury in vitro and in vivo was shown through the activation of NFkB/c-Rel, with a protective role during inflammation, thus highlighting the link between insulin/leptin, NF-kB, and ischemia [24].

Thus, it is worth mentioning that DMT1, responsible for NTBI, is known to be up-regulated through NF-kB-mediated epigenetic regulation in the aforementioned neurodegenerative diseases [8,9,10,11] and in neurodegeneration with brain iron accumulation (NBIA/BPAN), with the altered intracellular transport of iron and heavy metals found in primary fibroblasts of affected patients [5,25]. To this end, while considering iron’s influence on the homeostasis of CNS, previous studies have clearly described the brain distribution of the principal iron transporters, highlighting the important role of both the transferrin receptor (TfR) in the nervous system development [26], and divalent metal transporter-1 (DMT1). However, while TfR was shown to lack a complete match in the substantia nigra for the binding of transferrin in the quantitative ligand-binding autoradiography and densitometry of [125I]ferrotransferrin, with no difference between PD patients and control subjects for its contribution in the basal ganglia [27], the DMT1 up-regulation and iron increase were clearly described in the substantia nigra of post-mortem human brain slices, in the PD model of 1-methyl-4-phenyl-1,2,3,6-tetrahydropyridine (MPTP) intoxication in mice [8] and in the mice model of neurodegeneration with parkinsonism, the NFkB/c-Rel knockout mice [10], with the increased expression of DMT1 in both the substantia nigra pars compacta and striatum, and iron staining in the ventral tegmental area.

Furthermore, the NFκB-mediated epigenetic up-regulation of DMT1 was shown in the early phase of ischemic neurodegeneration, with ferrous iron and 1B/(−)IRE DMT1 accumulation found in the model of post-ischemic neurodegeneration during in vitro ischemia induced by oxygen–glucose deprivation (OGD) in primary mouse cortical neurons and the neuronally differentiated human neuroblastoma cells, SK-N-SH, where 1A/DMT1 showed no prevalent expression, with the up-regulation of the 1B/(−)IRE DMT1 protein and mRNA, also in mice subjected to transient middle cerebral artery occlusion (MCAO) [11]. Of note is the NFkB responsiveness of the 1B/DMT1 promoter during the early phase of neuronal ischemia through epigenetic regulation by acetylation at lysine 310 of NFkB/RelA; on the contrary, the abrogation of acetylation at lysine 310 of RelA determined the neuroprotection during OGD, with the down-regulation of both DMT1 protein and promoter transactivation, thus defining the pivotal role of the NFκB-dependent-1B/(−)IRE DMT1 isoform in the early phase of post-ischemic neurodegeneration, both in vitro and in vivo [9,11]. Again, the over-expression of the 1B/(−)IRE DMT1 isoform significantly increased the iron uptake in SK-N-SH, while the (−)IRE DMT1 knockdown by RNA silencing, in the same model, completely protected from both cell death and iron uptake during in vitro ischemia [11]. Accordingly, since insulin targets NFkB, known to exert the epigenetic regulation of DMT1 [6,9,11], responsive to inflammation and interleukins as shown in Langerhans cells [12], we produced evidence of neuroprotection by acute insulin treatment during in vitro ischemia induced by OGD in primary mouse cortical neurons and in differentiated human neuroblastoma cells with the concomitant down-regulation of both the DMT1 expression and ferrous iron uptake, thus focusing on the ferrous iron transporter DMT1 as a possible pharmacological target in the neuroprotection by insulin.

## 2. Material and Methods

***SK-N-SHcell culture. ***The human neuroblastoma cell line SK-N-SH was purchased from the American Type Culture Collection and differentiated into a neuronal-like phenotype by treatment with 50 µM retinoic acid (Sigma Aldrich, Milano, Italy) for 10–12 days, a condition known to induce mitotic arrest [28].

***Primary cultures of mouse cortical neurons*.** Embryonic mice at 15 days (C57Bl/6 dams, Charles River, Wilmington, MA, USA) were dissected to isolate cortical neurons and cultured for 10 days, as previously described [24], by the ARRIVE guidelines and with the U.K. Animals (Scientific Procedures) Act, 1986, and associated guidelines and Directive 2010/63/EU on the protection of animals used for scientific purposes.

***In vitro ischemia.***Oxygen–glucose deprivation (OGD) was performed as previously described [24]. Primary mouse cortical neurons at 10 DIV were subjected to OGD for 3 h, with or without 300 nM insulin (Sigma Aldrich, Milano, Italy, with 30 min pre-treatment with OGD. After OGD exposure in primary neurons, cellular lysis was followed by cell death evaluation, measuring the total LDH, the LDH release, and cellular content (Promega, Madison, WI, USA). SK-N-SH human neuronal cells, more resistant than cortical neurons to anoxia and noxious treatments like ferrous iron, were exposed to OGD for 8 h, with or without 300 nM insulin (Sigma Aldrich, Milano, Italy), with 30 min pre-treatment to OGD, and further analyzed for cell death by measuring the total LDH, the LDH released, and the cellular content (Promega, Madison, WI, USA).

***Ferrous iron treatment.*** After washes at neutral pH, differentiated SK-N-SH cells were incubated for 1 h in Hepes buffer at pH 6.0, in the presence or absence of 100 µM ferrous iron [29], with or without 300 nM insulin (SIGMA), and 30 min pre-treatment with OGD. After 1 h of treatment, the total LDH, LDH release, and cellular content were analyzed to determine the cell death parameter (Promega, Madison, WI, USA).

***Western blot analyses.***Total cellular extracts were prepared from differentiated SK-N-SH as described [9] for immunoblot staining with the following antibodies: rabbit anti-pan-DMT1 (H-108) and mouse anti-β-actin (C4) (Santa Cruz Biotechnology, Santa Cruz, CA, USA). Protein expression was normalized against housekeeping genes by densitometric analysis (Gel Pro Analyzer 3.0 analysis software).

***Reporter gene assay.***The activity of mouse 1B/DMT1 promoter was evaluated in the autologous cellular system of the mouse primary cortical neurons after 3 h OGD. The model of primary neurons reaches the plateau of cell death during OGD already at 3 h. Briefly, the cortical neurons were transfected at 10 DIV by LF 2000 Reagent (Thermo Fisher Scientific Inc., Waltham, MA, USA) with 0.2 mg/well of the DMT1-pGL3 plasmid, kindly provided by J.A. Roth [6], as described [30]. The transfection efficiency was normalized by co-transfection of 0.02 µg/well *Renilla* luciferase control plasmid (Promega, Madison, WI, USA). At 24 h after transfection, the neurons were subjected to 3 h of OGD, without or with 300 nM insulin (Sigma Aldrich, Milano, Italy), with 30 min pre-treatment with OGD. At the end of the OGD, the cells were harvested and both firefly and *Renilla* luciferase were analyzed using Dual-Luciferase Reporter Assay (Promega, Madison, WI, USA).

***Statistics.***All the results were expressed as the mean ± s.e.m. One-way ANOVA followed by Tukey’s post hoc analysis was applied to analyze differences between the groups. The statistical analysis was performed by Graph Pad Prism software (version 4.0, Boston, MA, USA) and differences were considered significant at the *p* value < 0.05.

## 3. Results

**Figure 1:**
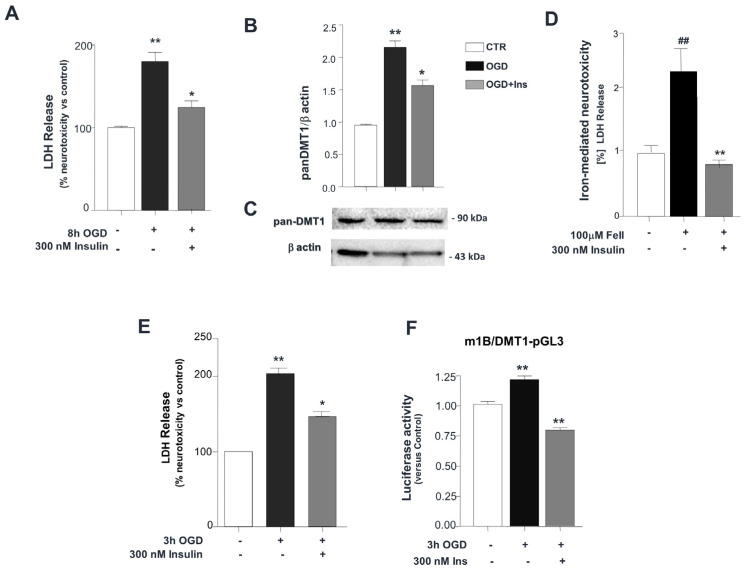
OGD- and ferrous-iron-dependent neurotoxicity are prevented by insulin in SK-N-SH neuronal cells and primary cortical neurons, through DMT1 down-regulation. (**A**) Cell death in differentiated neuronal SK-N-SH cells exposed to 8 h OGD, in the absence or presence of 300 nM insulin during OGD, with 30 min pre-treatment. Data represent the mean ± s.e.m. (percentage of control) for three independent experiments performed in triplicate. ** *p* < 0.001 vs. control; * *p* < 0.05 vs. control OGD. (**B**,**C**): DMT1 is up-regulated after 8 h of OGD with respect to control, in total cellular lysates of neuronally differentiated SK-N-SH, with down-regulation by 300 nM Insulin during OGD. (**B**) Densitometric analysis of pan-DMT1 reactivity as a ratio of relative β-actin level. The bars represent the mean of three separate experiments. ** *p* < 0.001 vs. control, and * *p* < 0.01 vs. control OGD. (**C**) A representative immunostaining is shown. (**D**) The neurotoxicity elicited by acute treatment with 100 μM ferrous iron for 1 h was significantly reduced in SK-N-SH cells after co-incubation with 300 nM insulin, and 30 min pre-treatment with OGD. Evaluation of cellular injury was determined by LDH release assay. The data represent the mean ± s.e.m. for three independent experiments performed in triplicate expressed as percentage values with respect to controls. ^##^ *p* < 0.001 vs. control, ** *p* < 0.001 vs. Fe(II) treatment. (**E**) Cell death evaluation in primary cortical neurons exposed to 3 h OGD. Treatment with 300 nM insulin was performed during OGD, with 30 min pre-treatment. The bars represent the mean ± s.e.m. for three separate experiments, performed in triplicate. (**F**) 1B/DMT1 promoter activity was evaluated in mouse primary cortical neurons at DIV 10 and the normalization of relative luciferase activity, after 3 h of OGD, was determined by measuring the co-transfection of the mouse 1B/DMT1 luciferase reporter plasmid (m1B/DMT1-pGL3) with the Renilla luciferase. The treatment with 300 nM insulin during OGD, with 30 min pre-treatment, down-regulated the OGD-induced promoter activity. The bars represent the mean ± s.e.m. for three experiments run in triplicate. ** *p* < 0.001 vs. control. Original images of (**C**) can be found in Supplementary Materials.

Treatment with 300 nM insulin, with a 30 min pre-treatment, in neuronal SK-N-SH cells exposed to 8 h of OGD significantly reduced both OGD-dependent cell death (** *p*< 0.001 vs. control; * *p* < 0.05 vs. control OGD) (Figure 1A) and the up-regulation of the glycosylated DMT1 component, compared to untreated cells (** *p* < 0.001 vs. control, and * *p* < 0.01 vs. control OGD) (Figure 1B,C). Furthermore, the neurotoxicity induced by acute incubation with 100 μM ferrous iron for 1 h, whose uptake at acidic pH is DMT1-dependent and mimics the ischemic-related extracellular milieu, was abrogated by a 300 nM insulin co-treatment, with a 30 min pre-incubation in SK-N-SH cells (^##^ *p* < 0.001 vs. control, ** *p* < 0.001 vs. Fe(II) treatment) (Figure 1D). In line with this evidence, in primary cortical neurons exposed to 3 h of OGD, the treatment with 300 nM insulin, with a 30 min pre-treatment with OGD, significantly protected against OGD-dependent cell death (** *p* < 0.001 vs. control, and * *p* < 0.01 vs. control OGD) (Figure 1E). Accordingly, the transactivation of the mouse 1B/DMT1 promoter was downregulated by acute treatment with insulin during the early phase of OGD, in the mouse cortical neurons (** *p* < 0.001 vs. control) (Figure 1F).

## 4. Discussion

DMT1 sustains the non-transferrin-bound iron transport (NTBI) with a finely tuned mechanism that maintains intracellular iron homeostasis. Alterations to this equilibrium can lead to iron accumulation, which is involved in several neurodegenerative diseases, such as post-ischemic neurodegeneration through the NF-kB-dependent up-regulation of DMT1, shown in both in vitro and in vivo models [9,11]. Due to the existing evidence of the insulin targeting of NF-kB signaling with protection from ischemic cell death in rat cardiomyocytes [22], we deemed the DMT1 regulation by insulin, previously found in Langerhans cells [12] and here described in the in vitro neuronal model of ischemic cell death, to be of possible translational interest.

Accordingly, the NF-kB-dependent role of DMT1 has been previously addressed in the early phase of post-ischemic neurodegeneration, in both differentiated human neuroblastoma cells (SK-N-SH) and primary cortical neurons exposed to OGD, and in the in vivo mice model of transient middle cerebral artery occlusion (tMCAO). Moreover, both DMT1 over-expression and (−)IRE/DMT1-isoform-specific RNA silencing significantly established the pivotal role of DMT1 in the same neuronal model of in vitro ischemia [11]. In this relationship, we here evaluated the effectiveness of insulin treatment in the protection by neuronal ischemic-dependent cell death with the contribution of DMT1 as a molecular target in both differentiated human neuroblastoma cells, SK-N-SH, and primary mouse cortical neurons, after 8 and 3 h of OGD, respectively (Figure 1A,E), where we found DMT1 down-regulation, after acute insulin treatment, at the protein level, in differentiated SK-N-SH after 8 h OGD compared to control OGD (Figure 1B,C) with significant neuroprotection against cell death during OGD (Figure 1A). In this relationship, in differentiated human neuroblastoma cells, SK-N-SH, the insulin treatment concomitant to acute ferrous iron exposure, performed in acidic conditions to promote maximum DMT1 uptake, showed greater neuroprotection from cell death, compared to the control treatment (Figure 1D). Moreover, in primary mouse cortical neurons, insulin treatment significantly protects from cell death and down-regulates the transactivation of 1B/DMT1 mouse promoter during OGD, already at 3 h, with respect to control OGD (Figure 1E,F).

Indeed, we found that insulin-dependent neuroprotection from cell death during neuronal ischemia is associated with the expression and function of DMT1, previously described as epigenetically regulated by NFkB, and the major ferrous iron transporter with a maximum uptake in acidic conditions as a proton co-transporter, particularly active during the extracellular acidosis present during ischemia. Interestingly, in this relationship, it has previously been shown that DMT1 over-expression in SK-N-SH human neuroblastoma cells leads to an increase in both cell death and intracellular iron content, while the knockdown of (−)IRE/DMT1 by siRNA completely prevented both iron uptake and cell death [11].

Although preliminary, these results show the neuroprotection of insulin targeting DMT1 expression and iron uptake during acidosis-dependent ischemic cell death, consequent to iron overload, as occurs in the pathogenesis of several neurodegenerative diseases. Interestingly, in this regard, the DMT1-associated NTBI [31] also has a role in beta-thalassemia major, one of the major iron-overload-related diseases, linked to metabolic impairment with insulin resistance and oxidative stress [32], thus sustaining an association between iron overload and glucose metabolism. The epidemiological studies of the association between iron overload and glucose metabolism showed the prevalence of type 2 diabetes in patients with hereditary hemochromatosis and iron-loading thalassemia, conversely explained by studies of glucose metabolism in the Belgrade rat model [33], which carries the point mutation G185R in the gene for DMT1, responsible for impaired iron uptake and consequent anemia, although associated with iron accumulation both in serum and liver. Of interest, in this model, is the aspect of a normal glycometabolic status in the absence of pancreatic damage, thus highlighting DMT1 loss as a protective factor against oxidative damage.

Importantly, the role of glucagon-like peptide-1 (GLP-1) receptor agonist Lixisenatide, an incretin mimetics drug, is already known in the treatment of diabetes, with established neuroprotection in rodent models of stroke, and Alzheimer’s and Parkinson’s disease [34], and its efficacy was recently confirmed in the treatment of motor disability described in a large cohort of patients with early Parkinson’s disease, in a phase 2, double-blind, randomized, placebo-controlled trial (https://clinicaltrials.gov/ ID NCT03439943 (accessed on 13 June 2018) [35]). Accordingly, these findings about the GLP-1 agonist further support the evidence of intranasal insulin neuroprotection in ischemic neurodegeneration [19,20,36], together with the clinical trials in Parkinson’s disease (NCT04251585,accessed on 4 February 2020; NCT05266417,accessed on 7 February 2022), that produced positive outcomes for both motor and cognitive impairment [37].

Thus, further studies are needed to analyze in greater detail the results we have highlighted here, concerning insulin neuroprotection during in vitro ischemia associated with DMT1 expression and function, and also the most recent evidence produced by the trials of both Lixisenatide and intranasal insulin in neurodegenerative diseases. The evidence highlights a consequent cross-sectional cascade of events that could support the neuroprotective role of insulin we found in the in vitro model of ischemic neurodegeneration through the regulation of NF-kB-dependent DMT1 expression and ferrous iron uptake.

We could indeed envisage a novel aspect for the involvement of ferroptosis in the glucose–neurodegeneration axis, found in several neurodegenerative diseases. However, further studies need to be carried out to reach conclusive results.

## Data Availability

Data are available upon request.

## Supplementary Materials

The following supporting information can be downloaded at: https://www.mdpi.com/article/10.3390/biom14070856/s1, Figure S1: Original images of Figure 1C.
