# Supplementary figures and images for: Ischemic Neuroprotection by Insulin with Down-Regulation of Divalent Metal Transporter 1 (DMT1) Expression and Ferrous Iron-Dependent Cell Death
Source: Biomolecules. 2024 Jul 15;14(7):856. doi: 10.3390/biom14070856 (PMC11274861; doi:10.3390/biom14070856)

Immunostaining rabbit anti-pan-DMT1

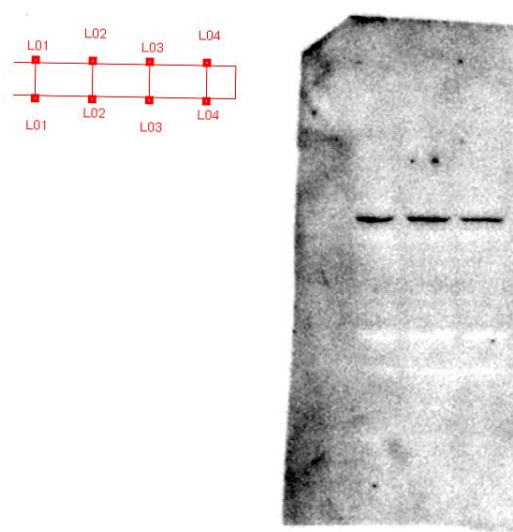

Immunostaining mouse anti-β-actin

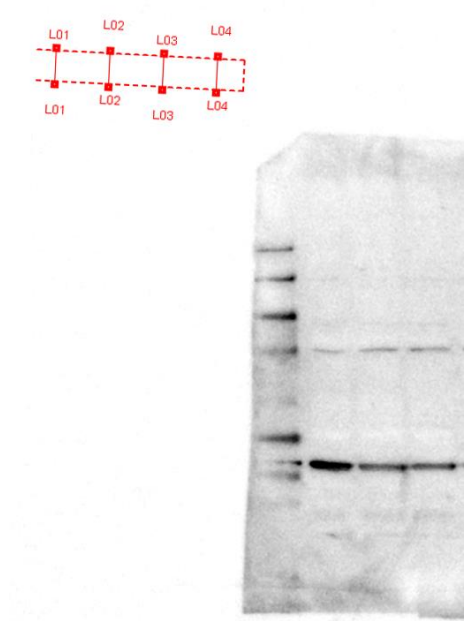

Supplement: Supplementary file 1 [file biomolecules-14-00856-s001.zip › biomolecules-3018539-supplementary.pdf]
